# Mutation Spectrum of *RB1* Gene in Unilateral Retinoblastoma Cases from Tunisia and Correlations with Clinical Features

**DOI:** 10.1371/journal.pone.0116615

**Published:** 2015-01-20

**Authors:** Hajer Ayari-Jeridi, Kimberly Moran, Amel Chebbi, Hédi Bouguila, Imen Abbes, Khaoula Charradi, Amel Benammar-Elgaaïed, Arupa Ganguly

**Affiliations:** 1 Laboratoire de Génétique, Immunologie et Pathologies Humaines, Faculté des Sciences de Tunis, Université de Tunis EL MANAR, Campus universitaire, Tunis, 2092, Tunisia; 2 Institut Hédi Rais d’Ophtalmologie de Tunis, Tunis, Tunisia; 3 Laboratoire d’Anatomie Pathologique, Institut Salah Azaiez de Cancérologie, Tunis, Tunisia; 4 Genetic Diagnostic Laboratory, Department of Genetics, Perelman School of Medicine, University of Pennsylvania, Philadelphia, Pennsylvania, United States of America; Centro de Investigación Príncipe Felipe – CIPF, SPAIN

## Abstract

Retinoblastoma, an embryonic neoplasm of retinal origin, is the most common primary intraocular malignancy in children. Somatic inactivation of both alleles of the *RB1* tumor suppressor gene in a retinal progenitor cell through diverse mechanisms including genetic and epigenetic modifications, is the crucial event in initiation of tumorigenesis in most cases of isolated unilateral retinoblastoma. We analyzed DNA from tumor tissue and from peripheral blood to determine the *RB1* mutation status and seek correlations with clinical features of 37 unrelated cases of Tunisian origin with sporadic retinoblastoma. All cases were unilateral except one who presented with bilateral disease, in whom no germline coding sequence alteration was identified. A multi-step mutation scanning protocol identified bi-allelic inactivation of *RB1* gene in 30 (81%) of the samples tested. A total of 7 novel mutations were identified. There were three tumors without any detectable mutation while a subset contained multiple mutations in *RB1* gene. The latter group included tumors collected after treatment with chemotherapy. There were seven individuals with germline mutations and all presented with advanced stage of tumor. There was no difference in age of onset of RB based on the germline mutation status. Thus 20% of the individuals with sporadic unilateral RB in this series carried germline mutations and indicate the importance of genetic testing all children with sporadic retinoblastoma. These findings help to characterize the spectrum of mutations present in the Tunisian population and can improve genetic diagnosis of retinoblastoma.

## Introduction

Retinoblastoma (*RB1*; OMIM 180200) is the most common intraocular neoplasm of childhood that originates from progenitors of retinal sensory cells with an estimated incidence of 1/20000 live birth, with a similar incidence rate in Tunisia.

The most common sign of this disorder is leukocoria, which is a visible white spot in the normally black pupil. Retinoblastoma (RB) has no significant predilection for gender or race. It is an early onset malignancy that usually occurs before the age of two years. In two-thirds of cases, the lesion is unilateral, afflicting one eye, and the median age of diagnosis is 2 years. In the other third, the lesion is bilateral, afflicting both, and the disease is diagnosed earlier, with a median age of diagnosis of 1 year. According to Knudson’s classic two-hit hypothesis, both alleles of the *RB1* tumor suppressor gene (GenBank accession number L11910), located on chromosome band 13q14.2 must be inactivated to initiate RB [1], [2].

Retinoblastoma occurs in two forms, hereditary (40%) and nonhereditary (60%). Hereditary disease, is caused by a constitutional mutation in *RB1* gene that predisposes to RB and other cancers later in life, and transmitted as an autosomal dominant trait with high penetrance (90%) [3]. In non-hereditary form, RB is initiated by two somatic mutations in *RB1* gene in retinal cells [4]. However, some rare forms of RB without mutations in *RB1* gene are associated with amplifications of MycN gene [5], [6], [7].

The majority of *RB1* mutations is unique to each family, and are distributed throughout the *RB1* gene including the promoter, coding exons, and splicing regions of introns [5], [8]. In addition, there is an excess of nonsense mutations arising from de-amination of methylated CpG di-nucleotides.

Early detection of children at risk to develop retinoblastoma is important to preserve life and vision. Therefore, the possibility of conservative management depends on early diagnosis [9]. Genetic testing has become an essential part of contemporary care for patients with retinoblastoma. Since the identification of the *RB1* gene in 1986 [10], there has been enormous progress in the methods used for identification of molecular defects in this gene. However, as the spectrum of oncogenic mutations in the *RB1* gene is very broad [11], a multi-step scanning protocol is needed that defines the order of the diverse analyses [5], [8], [12], [13], [14].

The aim for the present study was to identify the spectrum of mutations of *RB1* gene in 37 unrelated patients of Tunisian origin with unilateral retinoblastoma and to seek correlations between mutation status and clinical features.

## Patients and Methods

### Patients

Thirty-seven unrelated Tunisian patients with sporadic unilateral (36 patients) and bilateral retinoblastoma (1 patient without germline coding sequence alteration). All patients were recruited, examined and treated at the Institute of Ophthalmology Hedi Raies of Tunis, Tunisia. Diagnosis of RB was established by standard ophthalmologic and histological criteria. An overview of the clinical data for the patients is summarized in Table 1. Informed consent for mutation analyses was obtained for each case in accordance with the Declaration of Helsinki. The study was approved by the ethics committees of Pasteur Institute of Tunis and the Institutional Review Board of the University of Pennsylvania School of Medicine. As retinoblastoma is a childhood disease, written informed consent was obtained from the next of kin, caretakers, or guardians on behalf of the minors/children enrolled in this study.

**Table 1:** Spectrum of mutations in *RB1* gene identified in RB tumors from Tunisia.

**Samples**	**Gender**	**Laterality**	**Age at diagnosis (months)**	**Tumor Stage**	**Mutation Type**	**Site**	**Change in cDNA sequence**	**Consequence**	**Present in Blood**
*Tumors with two mutations in RB1 gene*									
RB 2	M	U	54	pT1	SP	Intron 12	c.1215+1G>A	Splicing	NO
					Intronic variation	Intron 25	c.2664–10T>A	….	NO
RB 6	F	U	54	pT2a	NS	Exon 9	**c.937G>T**	**p.Glu313X**	NO
					MS	Exon 21	c.2117 G>A	p.Cys706Tyr	NO
RB 13	F	U	11	pT2a	PM [HM 99,91%]	Promoter	…..	Silencing	NO
					LOH	…..	…..	…..	NO
RB 15	M	U	12	pT2a	PM [HM 92,65%]	Promoter	…..	Silencing	NO
					LOH	…..	…..	…..	NO
RB 22	M	U	24	pT3b	NS	Exon18	c.1735C>T	p.Arg579X	NO
					PM [HM 50%]	Promoter	…..	Silencing	NO
RB 31	M	U	20	pT1	NS	Exon 23	c.2359 C>T	p.Arg787X	NO
					LOH	…..	…..	…..	NO
RB 34	F	U	6	pT1	MS	Exon 26	**c.2665A>G**	**p.Lys889Glu**	NO
					LOH	…..	…..	**…..**	NO
RB 36	M	U	48	pT4	PM [HM 50%]	Promoter	…..	Silencing	NO
					Duplication	exon1, exon14 to exon16	…..	…..	NO
RB 38	F	U	24	pT3b	HEMIDEL	Whole *RB1* gene	No transcript	…..	NO
					LOH	…..	…..	…..	NO
RB 40	M	U	14	pT1	PM [HM 98,43%]	Promoter	…..	Silencing	NO
					LOH	…..	…..	…..	NO
RB 43	M	U	48	pT3a	Frameshift	Exon 3	**c.370–376dup**	**p.Ile126Asnfsx6**	YES **
					LOH	…..	**…..**	…..	NO
RB 49	M	U	8	pT3	HEMIDEL	Whole RB1 gene	No transcript	…..	YES
					NS	Exon19	**c.1901C>A**	**p.Ser634X**	NO
RB 51	M	U	36	pT2b	PM [HM 50%]	Promoter	…..	Silencing	NO
					LOH	…..	…..	…..	NO
RB 55	M	U	24	pT3b	NS	Exon 14	c.1363C>T	p.Arg455X	YES
					Frameshift	Exon 21	**c.2174DupT**	**p.Thr726AsnfsX24**	NO
RB 58	F	U	6	pT4	NS	Exon 18	c.1735C>T	p.Arg579X	NO
					LOH	…..	…..	…..	NO
RB 62	F	U	4	pT4	NS	Exon 11	c.1072C>T	p.Arg358X …..	YES
					HEMIDEL	Whole RB1 gene	NO transcript	…..	NO
RB 64	M	U	14	pT2	PM [HM 29,48%]	Promoter	…..	Silencing	NO
					LOH	…..	…..	…..	NO
RB 67	F	U	24	pT3b	NS	Exon 12	c.1150C>T	p.Glyn384X	NO
					LOH	…..	…..	…..	NO
RBX80	F	U	8	pT4	NS	Exon 17	c.1666 C>T	p.Arg556X	NO
					LOH	…..	…..	…..	NO
RBX101	M	U	20	pT1	NS	Exon21	c.2206C>T	p.Gln736X	NO
					LOH	…..	…..	…..	NO
RBX110	M	U	14	pT1	PM [HM 98,66%]	Promoter	…..	Silencing	NO
					LOH	…..	…..	…..	NO
RBX119	M	U	8	pT3	SP	Intron1	c.137+1G>A	Splicing	NO
					HEMIDEL	Whole RB1 gene	NO transcript	…..	YES
RBX137	F	U	24	pT3b	NS	Exon 12	c.1150C>T	p.Glyn384X	NO
					LOH	…..	…..	…..	NO
RBX109	M	U	12	pT3b	NS	Exon 10	c.958C>T	p.Arg320X	NO
					LOH	…..	…..	…..	NO
*Tumors with more than two mutations in RB1 gene*									
RB 7*	F	U	22	pT3	NS	Exon 10	**c.963C>G**	**p.Tyr321X**	NO
					NS	Exon 16	c.1494T>G	p.Thr498X	NO
					PM [IM 70%]	Promoter	…..	Silencing	NO
					LOH	…..	…..	…..	NO
RB 10*	F	U	8	pT4	NS	Exon 17	c.1666 C>T	p.Arg556X	NO
					PM [HM 35,42%]	Promoter	…..	Silencing	NO
					LOH	…..	…..	…..	NO
RB 20*	F	U	40	pT3b	NS	Exon 23	c.2359C>T	p.Arg787X	YES
					NS	Exon 18	c.1735C>T	p.Arg579X	NO
					MS	Exon20	**c.1975T>C**	**p.Tyr659His**	NO
					PM [IM 71,52%]	Promoter	…..	Silencing	NO
					LOH	…..	…..	…..	NO
RB 25	M	U	32	pT2a	NS	Exon 18	c.1735C>T	p.Arg579X	NO
					PM [HM 53,73%]	Promoter	…..	Silencing	NO
					LOH	…..	…..	…..	NO
RB 27	M	U	3	pT1	NS	Exon 17	c.1666C>T	p.Arg556X	NO
					SP	Exon 17	c.1695+1G>A	Splicing	NO
					PM [IM 99,24%]	Promoter	…..	Silencing	NO
RB 42*	F	U	18	pT3	NS	Exon 23	c.2359C>T	p.Arg787X	YES
					NS	Exon14	c.1333C>T	p.Arg445X	NO
					NS	Exon18	c.1735C>T	p.Arg579X	NO
					PM [HM 64,44%]	Promoter	….	Silencing	NO
*Tumors with one or no mutation in RB1 gene*									
RB 1*	M	U	48	pT3b	LOH	…..	…..	…..	…..
RB 5	M	U	12	pT1	LOH	…..	…..	…..	…..
RB 8	M	B	3	pT3b	LOH	…..	…..	…..	…..
RB 39	M	U	90	pT3b	LOH	…..	…..	…..	…..
RB 23*	F	U	44	pT3b	…..	…..	…..	…..	…..
RB 46*	M	U	42	pT3b	…..	…..	…..	…..	…..
RB 56*	F	U	6	pT3b	…..	…..	…..	…..	…..

Nucleotide numbering for complete coding sequence of DNA and cDNA are according to GenBank accession numbers L11910.1 and NM_000321.2, respectively. Amino acid numbering refers to the reference sequence for pRB protein, NP_000312.2.

MS, missense; FS, frameshift; NS, nonsense; SP, splicing; LOH, Loss of heterozygosity; PM, Promoter methylation; [HM], percentage of Hypermethylated fraction; [IM], percentage of intermediate methylated fraction.

Novel mutations are noted in boldface type.

(*) Patients treated with neoadjuvant chemotherapy.

(**) Patient with germline mosaicism.

(#) Patients with tumor samples used up before completing all the mutational analysis methods.

Majority of the mutation detection work was done in the Genetic Diagnostic laboratory, University of Pennsylvania, USA.

### DNA isolation

**Tumor samples.** Tumor DNA was isolated from fresh tumor tissue and paraffin embedded tissue sections using phenol/chloroform purification and The QIAamp DNAFFPE Tissue kit (QIAGEN GmbH, Hilden, Germany), respectively.

**Blood samples.** Total genomic DNA was isolated from EDTA treated peripheral blood using phenol/chloroform-based extraction. DNA integrity was evaluated by spectrophotometry at 260 and 280nm (Infinite 200 PRO NanoQuant, Tecan).

### *RB1* Promoter Methylation Analyses

The methylation status of the CpG island in the promoter region of the *RB1* gene was examined using EpiTect Methyl II PCR System kit (Qiagen, Valencia, CA, USA), following instructions of the manufacturer. In brief the method is as follows:

**Restriction digestion of tumor DNA.** A total of 250–500 ng of genomic DNA per sample was digested using the EpiTect Methyl DNA restriction kit according to the manufacturer’s instructions. Unmethylated and methylated DNA samples were digested by methylation-sensitive and/or a methylation-dependent restriction enzyme, respectively.

**Methylation qPCR.** Real-time PCR amplification of the *RB1* promoter region was performed using specific primers to discriminate methylated from unmethylated DNA and quantitated using SYBR Green qPCR Mastermix. DNA methylation levels were determined using the StepOne Software version 2.1 by calculating the percentages of unmethylated (UM), hypermethylated (HM) and intermediately methylated (IM) DNA.

### DNA sequence analysis of the *RB1* gene

The first phase of mutation analysis was performed on tumor DNA. The coding exons, flanking intronic regions, and the core promoter of the *RB1* gene were amplified by PCR using commercial PCR kits and automated thermocycler (PE 2700; Applied Biosystems [ABI]). When the DNA was obtained from fresh tissue or leukocytes, we were able to amplify all 27 exons via primers designed using Primer3 software (http://fokker.wi.mit.edu/primer3/). For DNA extracted from paraffin-embedded tissue sections, new primers allowing for smaller amplicons (less than 150 bp) were designed. PCR products were purified enzymatically using ExoI/SAP for removal of excess primers and dNTPs and subjected to Sanger sequencing using one of the PCR primers. Sequencing reactions were performed on an Applied Biosystem 3100 DNA analyzer using Big Dye Terminator kit (Applied Biosystems). Sequences were compared to the reference sequence of the *RB1* gene (GenBank L11910.1) using the Sequencher Aligner software (Sequencher 5.0. Gene Codes, Ann Arbor, MI, USA). Alterations of normal splice site were predicted by using bioinformatics tools (available at http://www.fruitfly.org/seq_tools/splice.html). Prediction of the damaging effects of novel missense mutations was performed using PolyPhen 2 bioinformatics tools (available at http://genetics.bwh.harvard.edu/pph2/).

### Detection of *RB1* large genomic rearrangement

In order to detect germline exonic deletions or insertions within the *RB1* locus, Multiplex Ligation-dependent Probe Amplification was performed with the SALSA MLPA probemix P047-B1 *RB1* available from MRC Holland (www.mrc-holland.com) following the manufacturer’s instructions. The data were analyzed using GeneMarker software version 1.9 (SoftGenetics, State College, PA, USA). Height ratios of fluorescent peaks lower or higher than the normal height ratio range of <0.7–1.3> were regarded as deletions and duplications of the targeted region, respectively.

### Loss of Heterozygosity (LOH) Analysis

LOH analysis was performed using multiplex PCR amplification of Short-tandem-repeat (STR) loci RBi2 and RBi20, located in intron 2 and in intron 20 of the *RB1* gene, respectively for comparing genomic DNAs isolated from tumor and peripheral blood. PCR products were analyzed on an ABI PRISM 3130 XL Sequencer. Data analysis was performed by GeneMarker software version 1.9 (SoftGenetics, State College, PA, USA).

**Statistical analysis.** The Fisher’s exact test was used to evaluate the significance of correlations between molecular data and clinical features. A p value less than 0.05 was taken as the level of significance.

**Mutation Nomenclature.** Mutations are described using the nomenclature system suggested by HGVS (www.hgvs.org/mutnomen). The *RB1* reference sequences L11910 (GenBank accession number) was used as a reference for genomic alterations. Data have been submitted to ClinVar database with accession number MDI-4417. Accession numbers for each mutation can be found in the S1 Table.

## Results

A total of 37 unrelated Tunisian patients with sporadic retinoblastoma were analyzed in this study, including 15 girls and 22 boys with an average age of 24 months. All cases are unilateral except one who presented with the bilateral form, without any germline coding sequence alteration. All available information is summarized in Table 1.

### *RB1* Somatic mutation spectrum

**Figure 1:**
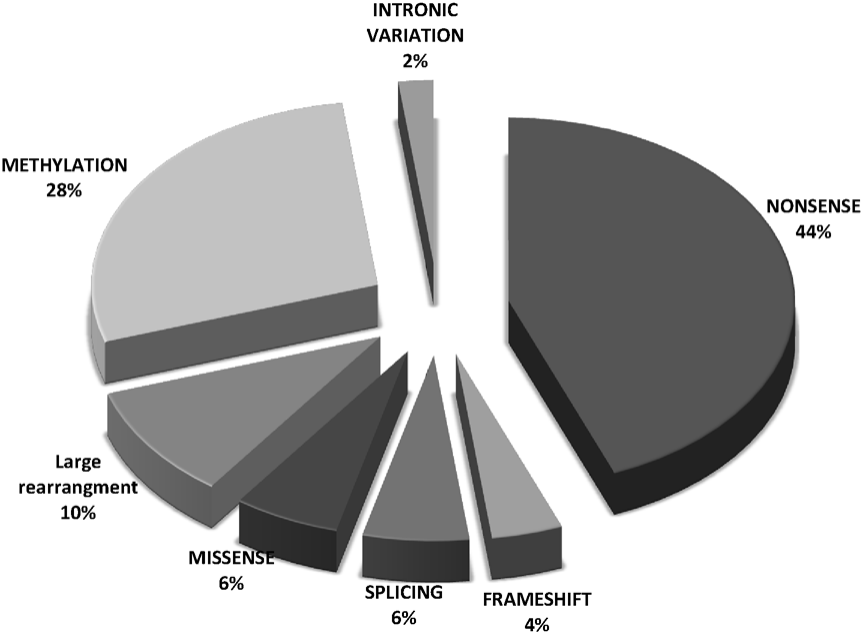
Frequency of somatic RB1 mutations in RB Tunisian patients (not including LOH).

Bi-allelic, inactivating *RB1* mutations were identified in 30 (81%) out of the 37 tumors tested (Table 1). A total of 50 somatic *RB1* alterations (excluding LOH) were identified, including point mutations and promoter methylations. Majority of the point mutations were nonsense mutations (22/50; 44%) followed by lower frequency of missense mutations (3/50; 6%), splice junction mutations (3/50; 6%), frameshift insertions (2/50; 4%), intronic variation (1/50; 2%). Aberrant promoter methylation (14/50; 28%), and large rearrangements (5/50; 10%) (Fig. 1) were observed.

Seven out of the 50 mutations (14%) were novel (http:// RB1-lovd.d-lohmann.de) and consisted of two missense mutations p.Tyr659His and p.Lys889Glu, and five null mutations that result in truncated pRB protein. The null mutations consisted of three nonsense substitutions [p.Glu313X], [p.Ser634X] and [p.Tyr321X], and two frameshift duplication [p.Ile126Asnfs*6] and [p.Thr726Asnfs*24] (Table1).

The frequency of LOH at the *RB1* locus was about 65% (24/37), representing the most frequent mutational event. It should be noted that, in addition to analysis of microsatellite markers flanking the *RB1* gene, LOH was also inferred when a mutation was identified in a hemizygous form that was revealed in the sequencing chromatograms with the presence of the mutant *RB1* allele only, and no evidence for the presence of the normal allele.

**Tumors with bi-allelic mutations in *RB1* gene.** Twenty-four (65%) tumors carried two mutations in *RB1* gene (Table1). Ten (42%) of these tumors carried coding sequence mutations combined with LOH. In 6 (25%) patients, two separate coding sequence alterations were identified, while 6 (25%) patients had LOH combined with *RB1*-promoter hypermethylation. The remaining two (8%) patients carried coding sequence abnormalities combined with *RB1*-promoter hypermethylation. Among these 24 tumors, loss of heterozygosity (LOH) was detected in a total of 16 (67%) cases.

**Tumors with more than two mutations in *RB1* gene.** In six tumor samples, molecular analysis identified more than two *RB1* mutations. The tumors could be divided into three groups: Group 1: characterized by three somatic events identified within RB10, RB25 and RB27 tumor samples. Group 2: characterized by four somatic events within RB7 and RB42 tumor samples. Group 3: up to five somatic events were identified within RB20 tumor sample. The majority of these accumulated events were null mutations, among which the same nonsense substitutions p.Arg787X, identified in RB20 and RB42 tumors, were present as germline mutations (Table 1). It must be noted that 4 (67%) of these 6 patients had undergone neoadjuvant chemotherapy before the tumors were collected.

**Tumors with only one or no inactivating mutation in *RB1* gene.** In tumors from 4 probands (11%), RB1, RB5, RB8, RB39, only one inactivating mutation was identified. In these tumors, the only mutation detected was loss of heterozygosity (LOH). Moreover, no mutations in the coding sequence of *RB1* gene were reported in an additional group of 3 tumors (RB23, RB46 and RB56). The DNA extracted from RB1 and RB23 tumor samples were insufficient for completion of all the mutation analysis steps.

### Germline mutations in *RB1* gene

To investigate the germline origin of any alteration observed in the tumor, the DNA isolated from the matched blood was screened for the presence of the mutations identified in the tumor. In 7 out of 36 sporadic unilateral RB patients (20%), one of the mutations identified in the tumor was also detected in germline DNA. These mutations included 4 nonsense mutations, 2 whole *RB1* gene deletions and 1 frameshift duplication leading to a premature stop codon. All these mutations were predicted to be null and result in haploinsufficiency of the pRB protein. Sanger sequencing in patients RB20, RB42, RB55 and RB62, showed that the ratio of signals of the normal and mutant alleles in DNA from blood was consistent with heterozygous C→T transition, at position c.2359, c.2359, c.1363 and c.1072 of the open reading frame (NM_000321.2), respectively. In contrast, using DNA isolated from blood of patient RB43, the signal of the mutant allele was significantly weaker compared to that from the normal allele. This is interpreted as evidence of mutational mosaicism indicating a mutation that occurred after conception [14], [15], [16].

### Correlations between molecular data and clinical features

**Figure 2:**
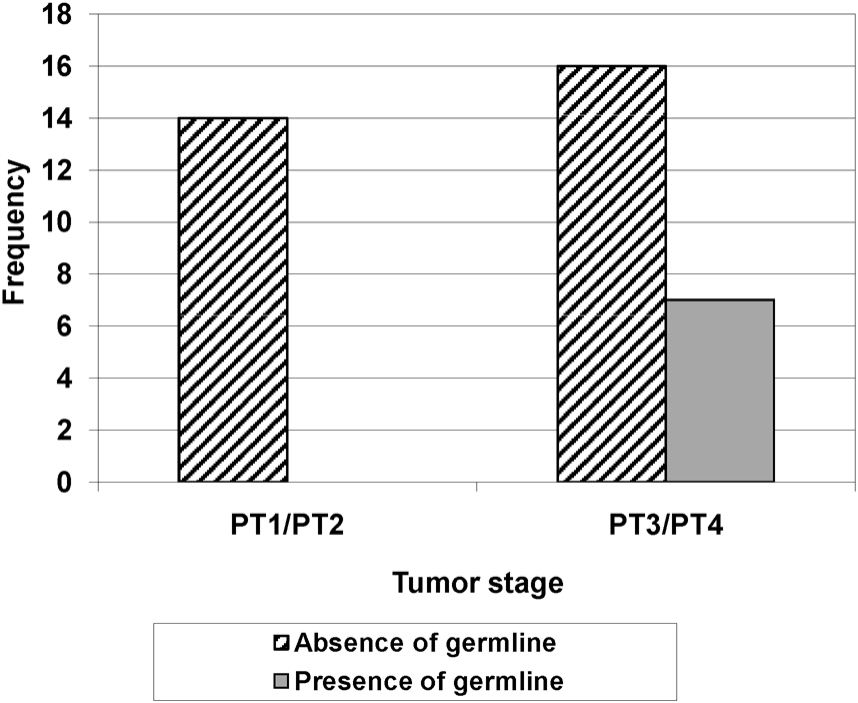
Correlation between tumor stage and presence or absence of germline RB1 mutation.

A correlation between tumors with and without LOH and the tumor stage was assessed. We noted that patients without LOH presented with more advanced tumor stage. We also compared the tumor stage in tumors derived from individuals with and without germline mutation and observed that all tumors derived from the former group are characterized by an advanced stage of tumor. The Fig. 2 illustrates this significant association (p = 0.031).

Next, we investigated tumor genotype with age at diagnosis. We observed that the distribution of ages at diagnosis in the 24 tumors with LOH and the 13 tumors without LOH are similar. In addition, the distribution of ages at diagnosis in the 7 individuals with and 30 individuals without a germline *RB1* mutation were assessed and no significant difference was observed.

The distribution of age at diagnosis is almost similar in both groups of patients with two or more *RB1* mutations median 21.5 months [4; 54] and 20.5 months [3; 40], respectively. The group of tumors with more than two mutations is characterized by an advanced tumor stage. On the other hand, tumors with one or without *RB1* mutation were characterized by a late age at diagnosis (median 35 months [3; 90]) and an advanced stage of tumor. All these results are summarized in Table 1 as well as in Table 2.

**Table 2:** Relationship between genotype and clinical features.

	**Number and % of probands**	**Median age at diagnosis**	**Stage of tumor**	
			**pT1/pT2**	**pT3/pT4**
*RB1* Somatic Mutational Status				
Patients with two mutations	24 (65%)	21.5 [4;54]	11 (46%)	13 (54%)
Patients with one or no mutations	7 (19%)	35 [3;90]	1 (14%)	6 (86%)
Patients with more than two mutations	6 (16%)	20.5 [3;40]	2(33%)	4(66%)
Constitutionnal *RB1* mutation				
Absent	30 (81%)	24,5 [3;90]	14(47%)	16 (53%)
Present	7 (19%)	21.4 [4;48]	0	7 (100%)
Loss of heterozygosity status				
LOH	24 (65%)	22.8 [3;90]	11 (46%)	13 (54%)
No LOH	13 (35%)	25.9 [3;54]	3 (23%)	10 (77%)

### Variants of unknown significance within *RB1* gene

The screening along the *RB1* gene sequence in somatic level identified 6 different variants of unknown significance (Table 3). Three out of these variations were novel, among which, a single nucleotide variation c.2523 T>G within exon 25 leading to silent mutation (T841T) was detected in 4 patients (11%). This variation seems to be frequent in the current series. In addition one silent variant, p.Ala628Ala, was detected in two tumors.

**Table 3:** Somatic variations of unknown signficance identified within *RB1* gene.

**Nucleotide position**	**Site**	**Allele 1**	**Allele 2**	**Reference**
**g.64497 G>A**	Int10	G	A	_
**g.65469 T>G**	Int 11	T	G	This study
**g.78378 A>T**	Int17	A	T	This study
**g.153277 A>G** *(c.1884) p.Ala628Ala*	Exon 19	A	G	This study
**g.156616 A>G**	Int19	A	G	_
**g.173709 T>G** *(c.2523) (p.Thr841Thr)*	Exon25	T	G	This study
**g.173882 G>T**	Int25	G	T	_

Nucleotide numbering for genomic DNA and cDNA are according to GenBank accession numbers L11910.1 and NM_000321.2, respectively. Amino acid numbering refers to the reference sequence for pRB protein, NP_000312.2.

## Discussion

Using an optimized multi-step mutation scanning protocol for tumor DNA [14], the spectrum of mutations in *RB1* gene was established in 37 unrelated Tunisian patients with sporadic retinoblastoma. This was followed by analysis of correlations of mutations with clinical manifestation of disease.

In the present study, hyper-methylation of the CpG island at the 5’ region of the *RB1* gene had been observed in 27% of cases. Such results reveal important involvement of *RB1* promoter hypermethylation in isolated unilateral RB in Tunisian population. This contrasts with the observations of Choy et al. 2002 in Chinese population, which showed that, in sporadic retinoblastoma, the major mechanisms inactivating *RB1* gene are commonly the loss of function mutations and loss of heterozygosity (LOH) but not the epigenetic events such as promoter hypermethylation [17].

In tumor RB2, one splicing alteration c.1215+1G>A in intron 12, was detected within the corresponding tumor. The second alteration, c.2664–10T>A, has been reported to be associated with RB [18]. These data indicated that this intronic variation was the second mutation inactivating *RB1* gene in RB2 patient.

Loss of heterozygosity (LOH) was found in 65% of the tumors, which is consistent with previous reports [19], [20] suggesting that loss of *RB1* allele plays an important role in tumor development. Furthermore, of 24 retinoblastoma tumors in which *RB1* bi-allelic inactivating mutations were identified, the second allele was mutated by LOH in 58% of tumors with nonsense substitution and in 75% of tumors with methylation of the promoter.

It is known, that mutations in *RB1*, that result in altered protein structure with partial retention of protein function, are over-represented in unilateral retinoblastoma [5], [21], [22]. These mutant alleles include mostly missense mutations, and also in-frame insertion/deletion mutations, a subset of splice-site mutations, or alterations in the promoter region.

In patient RB20, the novel missense mutation p.Tyr659His is located in the “B domain” which is part of “small pocket”, the most highly conserved regions of pRB throughout evolution and known to interact with many of the known pRb binding partners, including E2F transcription factors [23]. In addition, using the bioinformatic tool PolyPhen 2, p.Tyr659His was predicted to be damaging. The p.Lys889Glu, identified in RB34, was predicted to be benign; however we cannot exclude its deleterious effect on pRB functions given its location in the C-terminal domain that also interacts with E2F-DP complexes [24]. The novel silent variation, p.Ala628Ala, was detected in two individuals.

It has been known that approximately 15% of individuals with sporadic unilateral retinoblastoma, carry a germline mutation in *RB1* [25], [26]. These individuals can transmit retinoblastoma predisposition to their offspring. In order to improve disease management and familial planning, the germline or somatic origin of *RB1* alteration should be determined. In the Tunisian population, the proportion of isolated unilateral retinoblastoma patients 20% (7/36) who carry *RB1* mutant allele at constitutional level is higher than previous reports[15], [26]. However, this rate can be higher due to the phenomenon of germline mosaicism, which can hide post zygotic mutations [14], [15], [26], [27]. The high incidence of germline mutations confirms that the diagnosis of a unilateral sporadic case should prompt genetic screening and counseling.

According to Knudson’s classic hypothesis predicting that two hits are necessary to initiate Retinoblastoma, we anticipated finding bi-allelic somatic mutations within each one of the tumor samples. However, some tumors were characterized by presence of only one *RB1* mutation. It remains possible that the second alteration that is present in deep intronic regions or a rearrangement of the gene has been missed by the current assays [28], [14]. Moreover, in three tumors no *RB1* mutation was observed. These tumors can harbor amplification of the *MYCN* oncogene [7], altered pRB phosphorylation by PIN1 gene [29], or caused by viral infection [30].

There were 5 tumors characterized by the accumulation of somatic mutations, with more than two mutations in *RB1* gene. It must be noted, that 67% of these patients had undergone neoadjuvant chemotherapy. Therefore, the accumulation of these additional mutations in these tumors is a likely consequence of chemotherapy that damages DNA.

The associations between the mutations in *RB1* and age at diagnosis or tumor stage were analyzed. The early presentation of unilateral retinoblastoma is often interpreted as an indication of heritable disease [31]. However, in accordance with the observations of Lohmann et al., 1997 [26], the distribution of age at diagnosis was not different between patients with and without constitutional *RB1* gene mutation suggesting that age at diagnosis does not help to select carriers of germline *RB1* gene mutation among patients with isolated unilateral retinoblastoma. These data strongly support genetic testing of all children with RB irrespective of age at diagnosis.

All individuals with germline mutations presented advanced stages of tumor and were significantly different in the distribution of tumor stage in sporadic unilateral RB cases.

Our finding regarding the tumors without *RB1* mutations may contain amplification of the *MYCN* oncogene (MYCNA) according to the observation of Rushlow and colleagues [7].

This work helps to characterize the spectrum of somatic and germline mutations present in the sporadic unilateral RB population from Tunisia, and provide information documenting the importance of mutation screening of RB patients that can lead to improved molecular diagnosis and prediction of future risk of cancer in the proband and extended family.

## Supporting Information

S1 Table (XLSX)
